# Quantitative and Qualitative Analysis of the Antifungal Activity of Allicin Alone and in Combination with Antifungal Drugs

**DOI:** 10.1371/journal.pone.0038242

**Published:** 2012-06-05

**Authors:** Young-Sun Kim, Kyung Sook Kim, Ihn Han, Mi-Hyun Kim, Min Hyung Jung, Hun-Kuk Park

**Affiliations:** 1 Department of Obstetrics and Gynecology, School of Medicine, Kyung Hee University Hospital, Kyung Hee University, Seoul, Korea; 2 Department of Biomedical Engineering, College of Medicine, Kyung Hee University, Seoul, Korea; 3 Healthcare Industry Research Institute, Kyung Hee University, Seoul, Korea; 4 College of Oriental Medicine, Kyung Hee University, Seoul, Korea; 5 Program of Medical Engineering, Kyung Hee University, Seoul, Korea; Texas A&M University, United States of America

## Abstract

The antifungal activity of allicin and its synergistic effects with the antifungal agents flucytosine and amphotericin B (AmB) were investigated in *Candida albicans (C. albicans)*. *C. albicans* was treated with different conditions of compounds alone and in combination (allicin, AmB, flucytosine, allicin + AmB, allicin + flucytosine, allicin + AmB + flucytosine). After a 24-hour treatment, cells were examined by scanning electron microscopy (SEM) and atomic force microscopy (AFM) to measure morphological and biophysical properties associated with cell death. The clearing assay was conducted to confirm the effects of allicin. The viability of *C. albicans* treated by allicin alone or with one antifungal drug (AmB, flucytosine) in addition was more than 40% after a 24-hr treatment, but the viability of groups treated with combinations of more than two drugs was less than 32%. When the cells were treated with allicin alone or one type of drug, the morphology of the cells did not change noticeably, but when cells were treated with combinations of drugs, there were noticeable morphological changes. In particular, cells treated with allicin + AmB had significant membrane damage (burst or collapsed membranes). Classification of cells according to their cell death phase (CDP) allowed us to determine the relationship between cell viability and treatment conditions in detail. The adhesive force was decreased by the treatment in all groups compare to the control. Cells treated with AmB + allicin had a greater adhesive force than cells treated with AmB alone because of the secretion of molecules due to collapsed membranes. All cells treated with allicin or drugs were softer than the control cells. These results suggest that allicin can reduce MIC of AmB while keeping the same efficacy.

## Introduction

*C. albicans*, a systemic fungus, is a major cause of morbidity and mortality in patients immunocompromised as a result of AIDS, cancer chemotherapy, radiotherapy, organ transplantation, or bone marrow transplantation [1]–[2]. To treat *C. albicans* infections, the antifungal agents flucytosine and amphotericin B (AmB) are conventionally used in a clinical setting. However, treatment with these agents can cause severe side-effects, especially in immunocompromised patients or those who receive repeated dosing to treat recurrent infections. It is preferable to prevent fungal infections in high-risk patients rather than having to treat them. The primary preventive method against fungal infection is good hygiene, such as keeping the skin clean and dry. Several alternative medicines have gained popularity for the safe and effective prevention of fungal infections. Extracts from several natural sources have also been shown to have antifungal activity, including those from *Euphorbia hirta* L [3], *Eqoul* [4], *Tribulus terrestris* L [5], and allicin from garlic [6]–[16].

Among these, allicin has been the most actively investigated because of its prominent antifungal effects. Allicin is an organic compound, derived mainly from garlic, which contains sulfur. When garlic is crushed or damaged, alliin, which exists naturally in garlic, reacts with the enzyme allinase. Allinase acts as a catalyst to transform alliin into allicin (diallyl thiosulphinate). Several studies have demonstrated that pure allicin has strong anti-bacterial and anti-fungal properties [6]–[16]. Allicin inhibited both the germination of spores and the growth of hyphae produced by *Candida, Cryptococcus*, and *Trichophyton* species [6]–[9]. The concentrations of ß–lactam antibiotics that inhibited the growth of *Staphylococcus spp*. and *Pseudomonas aeruginosa* were reduced in the presence of allicin [10]–[11]. Allicin has also been shown to increase oxidative stress, reduce glutathione levels, and inhibit biofilm formation in *C. albicans* [12]–[4]. The antimicrobial effects of allicin are related to the ability of allicin to strongly inhibit thiol-containing enzymes such as cysteine proteinases, alcohol dehydrogenases, and thioredoxin reductases [15]. In addition, it was recently suggested that allicin could increase the activity of Cu^2+^, which is a known promoter of antimicrobial activity; allicin works by accelerating the production of endogenous reactive oxygen species (ROS) [16].

Although, allicin has marked antimicrobial effects, it has limited clinical applications, because the minimal inhibitory concentration (MIC) of allicin is relatively high [17]. Therefore, allicin is used mainly as a supplemental agent to enhance the efficacy of chemical agents. Our goal in this study was to quantitatively and qualitatively investigate the antifungal activity of allicin alone and in combination with antifungal drugs. Our results suggest that allicin can be used to decrease the doses of antifungal agents required to inhibit *C. albicans* growth. Especially, it was observed significant synergistic effects when allicin used in conjunction with AmB. By measuring the changes in morphology and biophysical properties of *C. albicans* treated with allicin by scanning electron microscopy (SEM) and atomic force microscopy (AFM), the antifungal effects of allicin was analyzed quantitatively. By clearing assay, the effects of allicin were clearly visualized. In addition, we investigated the activity of allicin in combination with the antifungal agents, flucytosine and AmB.

## Results

**Figure 1:**
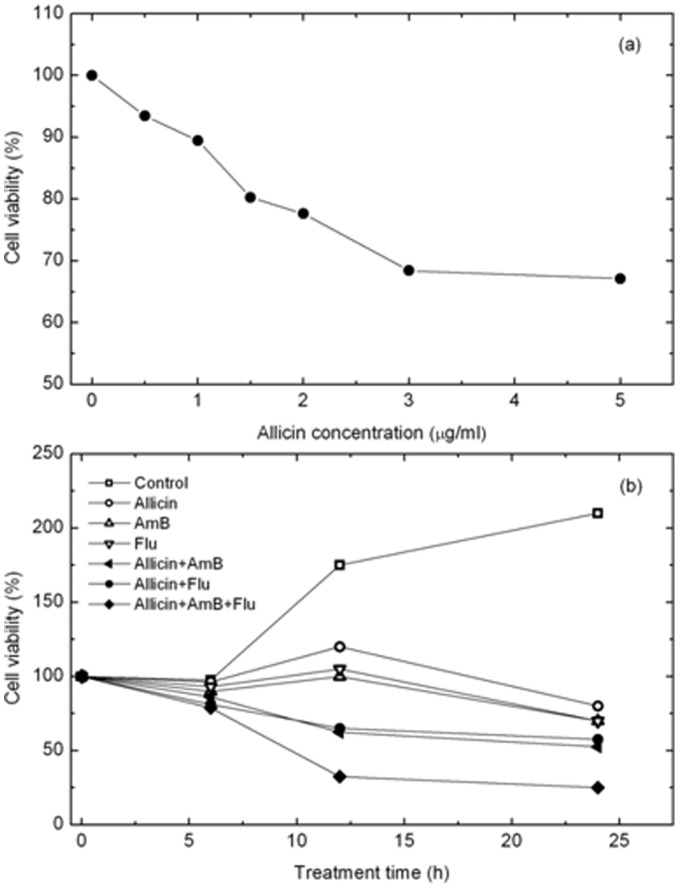
Cell viability of *C. albicans* treated with allicin and antifungal drugs. (a) The viability of *C. albicans* as a function of allicin concentration. The concentration of allicin was increased from 0 (control) to 5 µg/mL. (b) Cell viability as a function of treatment conditions. *C. albicans* cells were treated with allicin alone, one kind of drug (AmB, flucytosine), and the combinations of AmB + allicin, flucytosine + allicin, and AmB + flucytosine + allicin.

The antifungal activities of allicin alone and in combination with AmB and flucytosine were estimated using a cell viability assay. Figure 1(a) shows *C. albicans* viability at various concentrations of allicin ranging from 0 (control) to 5 µg/mL. Cell viability decreased as the allicin concentration increased, but the rate of reduction was not significant. When the allicin concentration increased 10-fold from 0.5 to 5 µg/mL, the reduction in cell viability was only 24%. The MIC_10_ of allicin for *C. albicans* was therefore determined to be 1 µg/mL. Figure 1(b) shows cell viability as a function of drug treatment time from 0 (control) to 24 hours with different drugs. The viability of cells treated by allicin alone or one of the antifungal drugs (AmB, flucytosine) was relatively high (more than 40% at 24 hr), but that of cells treated with combinations of allicin and the two drugs (AmB + allicin, flucytosine + allicin, and AmB + flucytosine + allicin) was relatively low (less than 32% at 24 hr). In all cases, cell viability showed a similar dependence on treatment time; viability decreased rapidly after 6–12 hours and then gradually decreased with longer treatment. In particular, colonies treated with AmB + allicin and flucytosine + allicin showed the same viability for all treatment conditions. The slight increase in viability observed at 24 hours is due to the budding of new cells from undamaged cells.

**Figure 2:**
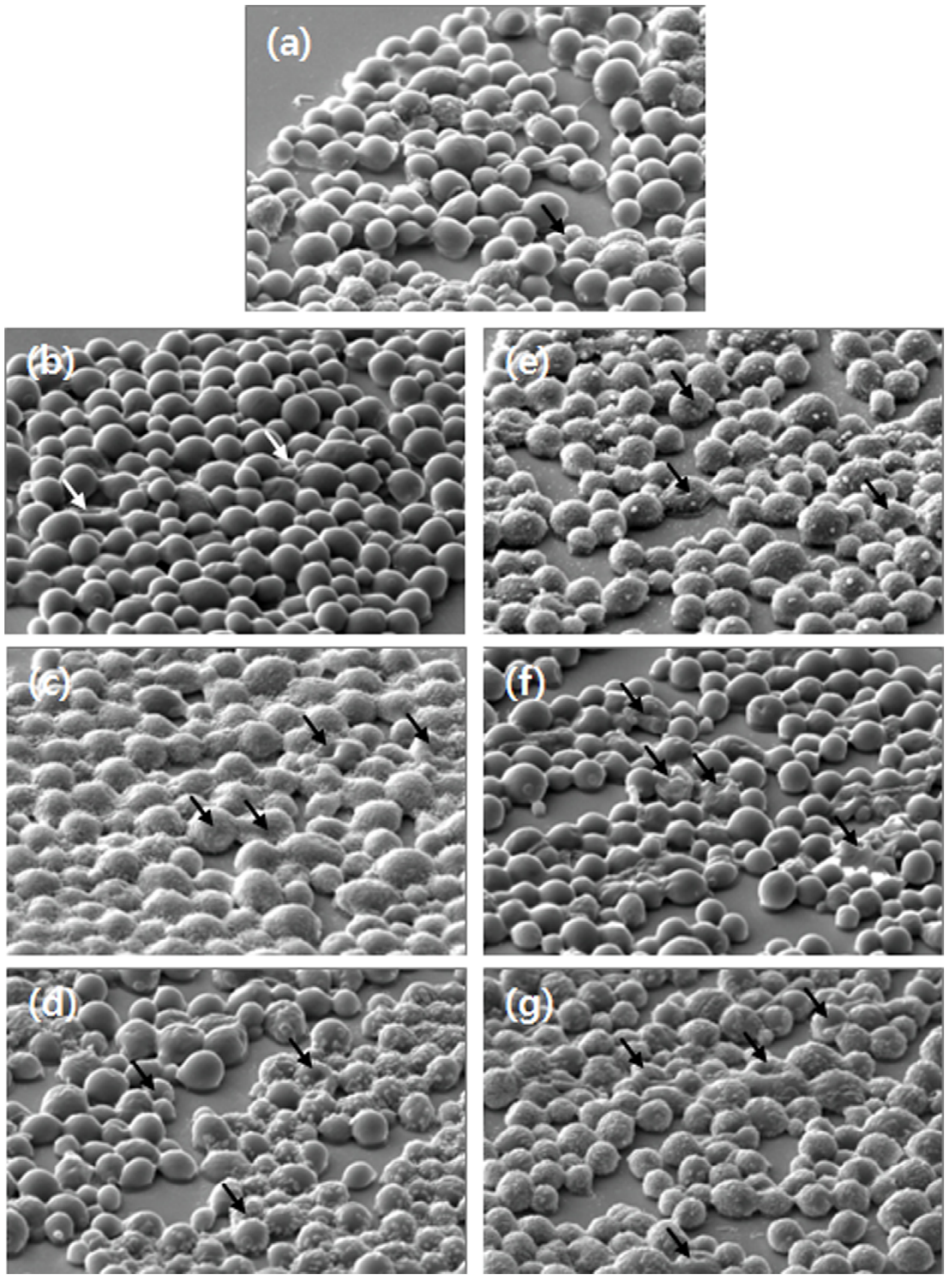
SEM images of *C. albicans* treated with allicin or antifungal drugs for 24 hours. (a) Control cells. The images shown in (b)–(g) are of cells treated with allicin alone, AmB, flucytosine, AmB + allicin, flucytosine + allicin, and AmB + flucytosine + allicin, respectively.

The effects of the various antifungal drugs on the morphology of *C. albicans* were investigated by two spectroscopic techniques, namely SEM and AFM. Using SEM images, overall changes in the morphology of the cells were monitored in the relatively large area of 50×50 µm^2^. Figures 2(a)–(d) show images of untreated *C. albicans* (control) (a), or *C. albicans* treated with AmB (b), flucytosine (c), or allicin (d), respectively. Figures 2(e)–(g) are images of cells treated with the combinations of AmB + allicin (e), flucytosine + allicin (f), and AmB + flucytosine + allicin (g). All cells were treated for 24 hours. No significant morphological changes, such as deformation or shrinkage, were observed in any of the treatment groups based on SEM images.

**Figure 3:**
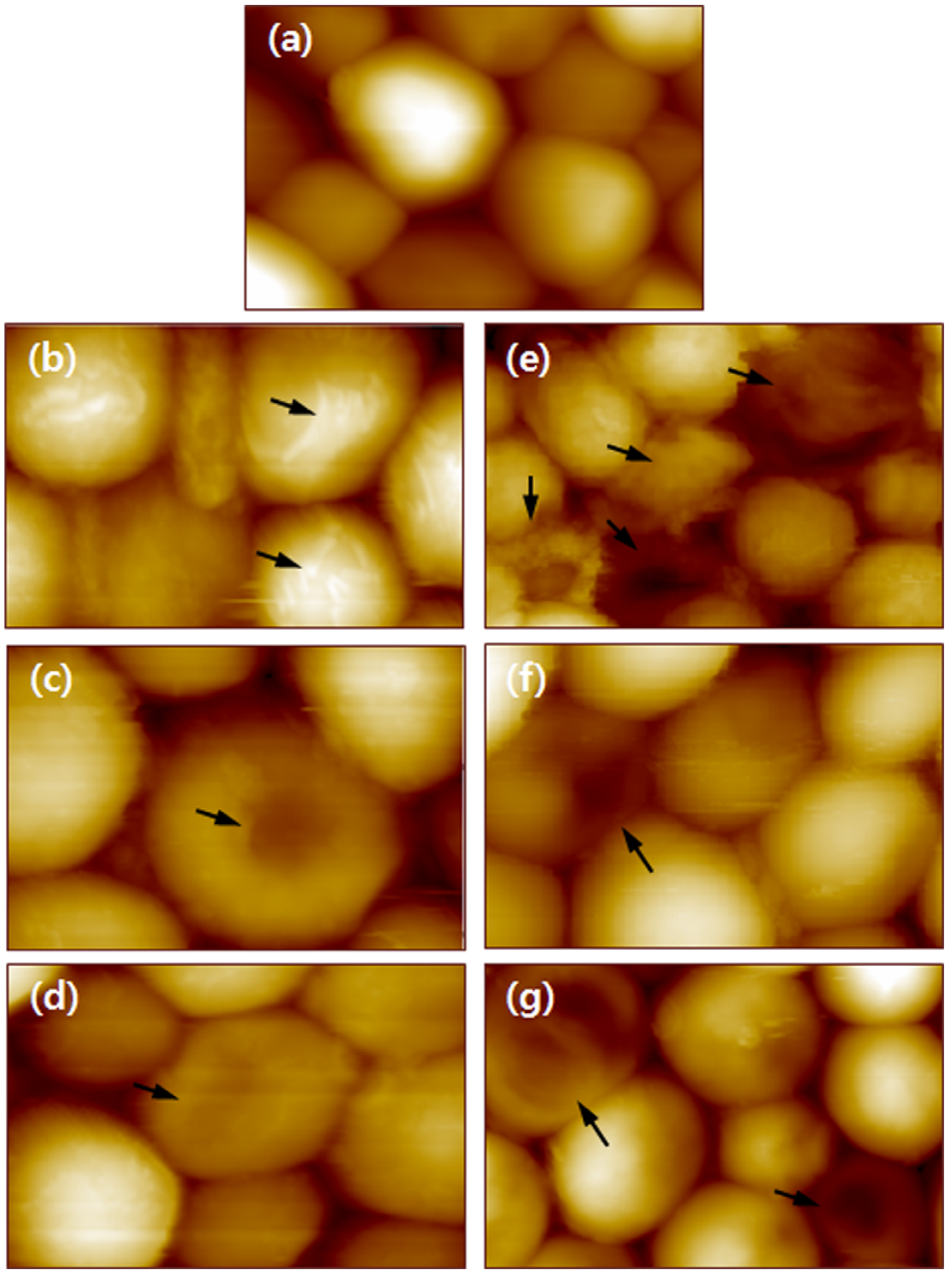
AFM images of *C. albicans* treated with allicin or antifungal drugs for 24 hours. (a) Control cells. The images shown in (b) – (g) are of cells treated with allicin alone, AmB, flucytosine, AmB + allicin, flucytosine + allicin, and AmB + flucytosine + allicin, respectively. The arrows indicate significant morphological changes.

AFM images revealed morphological changes of *C. albicans* more clearly than the SEM images because AFM is extremely high-resolution type of scanning probe microscopy with a demonstrated resolution in the sub-nanometer range. To increase the resolution more, all images were measured at the very low scan speed of 0.2 lines/sec. Representative AFM images of *C. albicans* are shown in Figure 3, and the figures are arranged in the same manner as in Fig. 2. Untreated cells (Fig. 3a) were intact and had smooth surfaces. Cells treated with AmB showed some peeling of the outer membrane (Fig. 3b), while cells treated with flucytosine had collapsed outer membranes (Fig. 3c). In addition, cells treated with allicin had collapsed membranes (Fig. 3d), but the changes were not significantly different compared to cells treated with flucytosine. Cells treated with the combination of allicin and AmB or flucytosine showed more damage than expected (Figs. 3e–g). In particular, the membranes of *C. albicans* treated with AmB + allicin appeared as if they had burst or collapsed (Fig. 3e).

**Figure 4:**
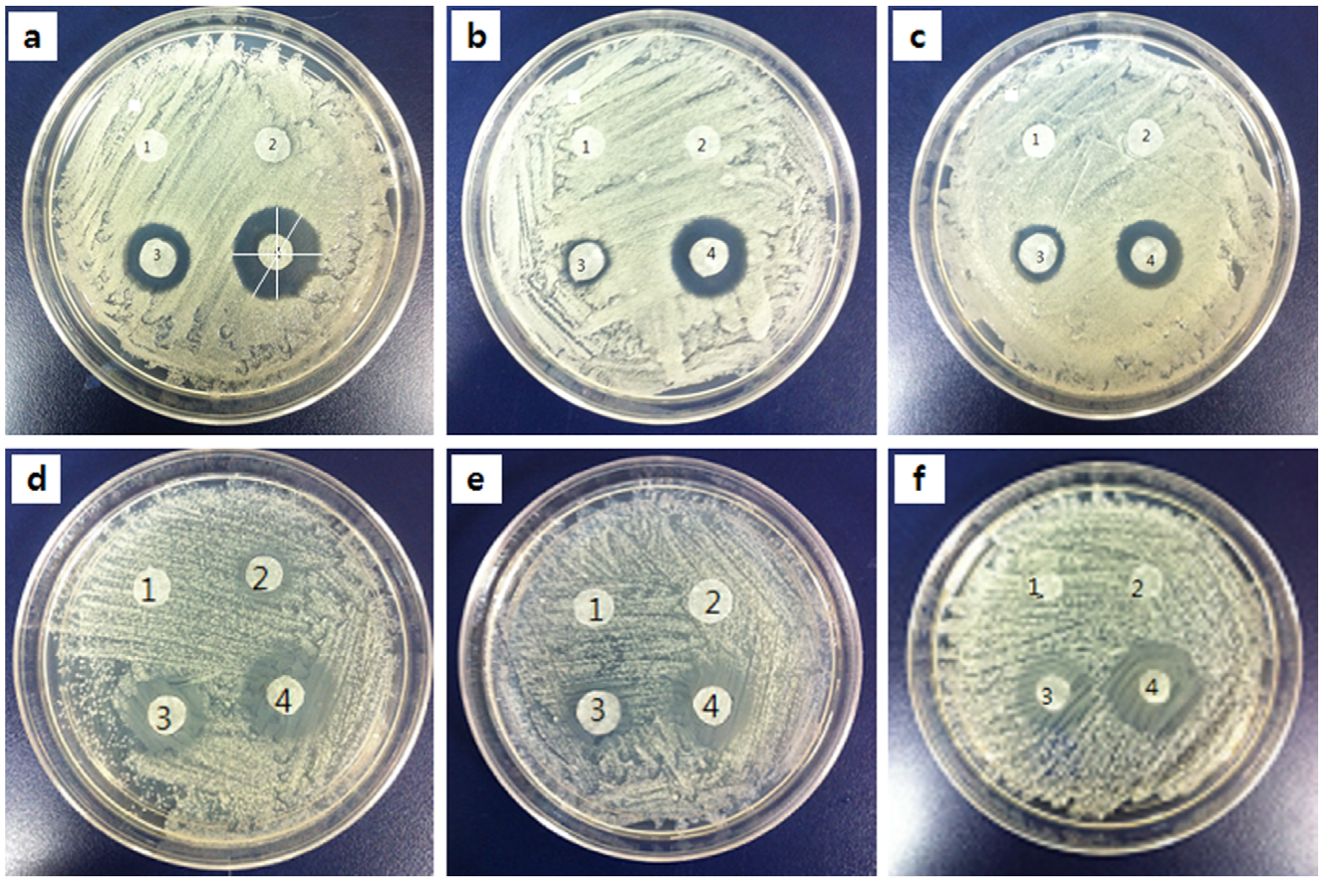
Clearing assays with AmB (a), flucytosine (b), allicin (c), AmB + allicin (d), flucytosine + allicin (e), and AmB + flucytosine + allicin (f). Spot number 1 indicates the control sample. The spots numbered 2, 3, and 4 correspond to antifungal treatments with the concentrations of MIC_10_×10^−1^, MIC_10_, and MIC_90_, respectively.

We also evaluated the effects of allicin and the two antifungal agents against *C. albicans* quantitatively. The results of a clearing assay with AmB (a), flucytosine (b), allicin (c), AmB + allicin (d), flucytosine + allicin (e), AmB + flucytosine + allicin (f) are shown in Fig. 4. The assay was conducted with serial dilutions of MIC_10_×10^−1^, MIC_10_, and MIC_90_, respectively. In all plates, the growth inhibitory zone around specific antifungals increased as the concentration of antifungal agent increased. The size of clearance zone was measured and the result was summarized in table 1. Since the clearance zone is not a perfect circle, diameter of the zone was measured at several directions as indicated in Fig. 4(a). Then, the values were averaged.

**Table 1:** The diameter of clearance zones according to treatment condition.

	Diameter of clearance zones (cm)			
Spot number(Concentrations)	1(Control)	2(MIC_10_×10^−1^)	3(MIC_10_)	4(MIC_90_)
Amphotericin B		0.487±0.012	0.926±0.047	1.236±0.053
Flucytosine			0.653±0.018	1.083±0.058
Allicin			0.757±0.041	0.931±0.029
Amphotericin B + Allicin		0.727±0.047	1.067±0.012	1.282±0.012
Flucytosine + Allicin			0.931±0.006	1.166±0.105
Amphotericin B + Flucytosine +Allicin			1.005±0.017	1.36±0.047

The cell death phase (CDP) of *C. albicans* was analyzed by using SEM and AFM images. The CDP was divided into four steps (CDP_0_, CDP_1_, CDP_2_, and CDP_3_) according to morphological changes [18]. The CDP results are listed in Table 2; these results were obtained by counting the number of cells after a 24-hour treatment. Because the drug concentration was low (MIC_10_), many cells in all groups were at CDP_0_. Even though cell viability did not vary much according to the type of drug as shown in Fig. 1, the detailed CDP of the cells was very different according to the treatment condition. The result of CDP shows a good agreement with the clearing assay.

**Table 2:** The normalized number of cells at different cell death phases (CDP) according to treatment condition.

	Normalized number of cells (%)			
Treatment condition	CDP_0_	CDP_1_	CDP_2_	CDP_3_
Control	93.61±4.47	1.68±2.38	2.84±1.54	1.85±0.55
Amphotericin B	70.49±5.86	5.47±0.97	13.57±0.24	10.46±4.64
Flucytosine	71.16±4.69	7.44±2.94	1.90±0.07	3.88±1.66
Allicin	79.45±3.92	5.83±3.22	8.38±3.23	6.32±3.91
Amphotericin B + Allicin	49.97±12.23	13.43±4.37	18.53±1.83	18.05±6.02
Flucytosine + Allicin	72.49±12.70	10.22±2.99	7.93±5.98	9.34±9.72
Amphotericin B + Flucytosine +Allicin	52.51±2.60	9.86±6.78	24.07±3.87	13.55±0.31

The CDP was assessed by analyzing SEM and AFM images of cells treated for 24 hours.

**Figure 5:**
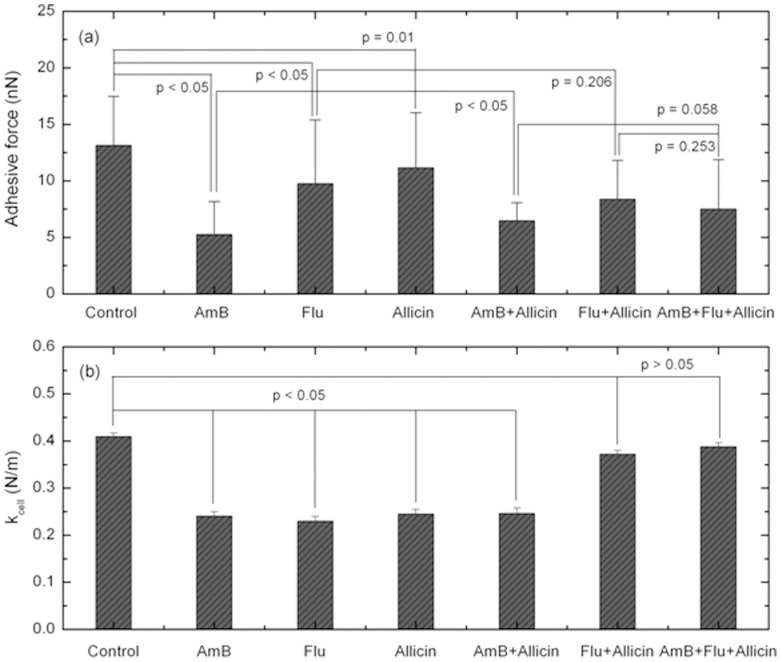
Changes in the biophysical properties of *C. albicans* according to treatment condition. (a) Changes in the adhesive force between the cells surface and the AFM tip and (b) changes in the stiffness of the cell membrane.

Changes in the biophysical properties of *C. albicans* induced by allicin or antifungal drug treatment were investigated by force-distance (FD) curve measurements using AFM. The adhesive force and stiffness results obtained from analyzing FD curves are shown in Figs. 5(a) and (b), respectively. There were obvious changes in adhesive force according to the treatment conditions. The adhesive force of all cells treated with allicin or drugs was decreased compared to that of control cells. However, the amount the adhesive force was reduced by differed with treatment condition. The adhesive force of cells treated with AmB and flucytosine was significantly lower than that of control cells. However, cells treated with allicin showed only a slight decrease in adhesive force. Cells treated with AmB + allicin had a higher adhesive force than cells treated with AmB alone. However, in the cells treated with flucytosine + allicin, the adhesive force was slightly decreased compare to that of cells treated with flucytosine alone. Cells treated with AmB + flucytosine + allicin showed no noticeable changes in adhesive force compared to the other groups. All cells treated with allicin or drugs were softer than the control cells. When the cells were treated with the combination of flucytosine + allicin, the stiffness of the cells increased compared to that of cells treated with flucytosine alone.

## Discussion

In this study, we analyzed the antifungal effects of allicin quantitatively and qualitatively by measuring the changes in morphology and biophysical properties of *C. albicans*. In addition, we evaluated the activity of allicin in combination with AmB and flucytosine. *C. albicans* cells were not seriously damaged when treated with allicin alone. However, when allicin was used in combination with antifungal drugs, the cells were seriously damaged or destroyed. In particular, the cells treated with AmB + allicin were more damaged than the cells treated with flucytosine + allicin. The outer membranes of cells treated with AmB + allicin were completely destroyed and the adhesive force of these cells was higher than that of control cells.

Allicin has been reported to have antibacterial, antifungal, antiparasite, and antiviral activity [6]–[16]. A broad range of bacteria, including *E. coli, Staphylococcus aureus*, *Streptococcus pyogenes*, *Proteus mirabilis*, *Pseudomonas aeruginosa*, *Acetobacter baumanii*, *Klebsiella pneumoniae*, *Enterococcus faecium*, *Myco-bacterium tuberculosis*, *H. pylori, Salmonella*, *Clostridium,* and *Shigella* show allicin sensitivity. Although allicin is a very useful natural compound for treating fungal infections, its high MIC prevents its effective use in a clinical setting. Pure allicin has been shown to have antifungal activity against species of *Candida*, *Cryptococcus*, *Trichophyton*, *Epidermophyton*, and *Microsporum* at concentrations of 1.57 ∼ 6.25 µg/mL [17]. These are very high concentrations compared to those of antifungal drugs; for example, the MIC_50_ and MIC_90_ of AmB against *C. albicans* is 0.5 and 1.0 µg/mL, respectively [19]. One garlic bulb contains 6 ∼ 14 mg/g of alliin, which is transformed into allicin by allinase [20]. Therefore, it will be more effective to use allicin in combination with conventional antifungal agents rather than allicin used alone. In this manner, the efficacy of conventional drugs can be enhanced with the minimal concentrations.

The synergic effects of allicin was investigated with two drugs of AmB and flucytosine, which used only a small amount (MIC_10_) to detect from the early stage to the final stage of the cell death process. There was no difference in the cell viability of the cells treated with AmB + allicin and the cells treated with flucytosine + allicin. However, when cells were characterized according to the CDP, we found that there were more cells at CDP_0_ in the group treated with flucytosine + allicin than in the group treated with AmB + allicin. However, there were more CDP_1_ cells in the group treated with AmB + allicin than the group treated with flucytosine + allicin, and this difference increased at CDP_2_ and CDP_3_. As shown in the AFM images, the cells treated with AmB showed changes in the outer membrane, and were seriously damaged when allicin was added [21]. These results indicate that the antifungal efficacy of AmB is significantly enhanced in the presence of allicin. This result can be understood by considering the mechanisms of action of both AmB and allicin. AmB is thought to bind to ergosterol and then destroy the integrity of the fungal membrane [22]. In addition, AmB is thought to induce ROS-based oxidative damage [23]. Correlated with this mechanism, it was recently reported that allicin could increases the antifungal activity of Cu^2+^ [16]. Cu^2+^ is toxic to living cells because it accelerates the generation of ROS. Living cell membranes are damaged by ROS that oxidize proteins; in addition, ROS can damage DNA and RNA. Akiar et al. reported that the lethal effects of Cu^2+^ were significantly greater in the presence of allicin [15]. The antifungal activity of AmB was likely enhanced by allicin because allicin increased the permeability of the cell membrane. In contrast, the efficacy of flucytosine was not much improved by allicin co-treatment in the context of morphological changes. This result can be explained by considering flucytosine’s mechanism of action. Flucytosine acts mainly on the RNA and DNA of fungi. Flucytosine alters the amino-acylation of tRNA and disturbs the building of essential proteins due to its incorporation into fungal RNA. It also inhibits fungal DNA synthesis after it is converted into 5-fluorodeoxyuridinemonophosphate [24]. Cells treated with this antifungal agent were damaged initially from the interior, as indicated by membrane collapse. Flucytosine and allicin act on different parts of the cell and have different mechanisms, and therefore show no synergistic effects.

The adhesive force is the strength of the interaction between the cell membrane and the AFM tip, and it is very sensitive to the properties of the membrane. Cells treated with flucytosine had less adhesive force than control cells, consistent with the results reported in our previous study [18]. While, the cells treated with AmB showed an opposite trends to our previous results, the adhesive force was increased by the treatment with AmB as in a previous study, but was decreased in this work [18]. Adhesive force generally decreases during cell death [25]. In the previous study, we assumed that the increased adhesive force measured after treatment of cells with AmB was due to secretion that occurred after destruction of the cell membrane and not due to an interaction between the cell membrane and the AFM tip. The decreased adhesive force of cells treated with AmB demonstrates that our assumption was correct. As shown in Fig. 3(b), because the concentration of AmB was low (MIC_10_), most cells were at CDP_0_ while the other cells just started showing morphological changes. AmB treatment did not result in the destruction of the cell membrane, and therefore the adhesive force of these cells decreased as was observed for the other treated cells. However, when cells were treated with both AmB and allicin, the membranes of the cells burst, as shown in Fig. 3(e), resulting in secretion of internal molecules. This increased the adhesive force of these cells compared to cells treated with AmB alone.

In conclusion, allicin has antifungal activity but a very high MIC against pathogenic fungi. However, the antifungal activity of the conventional drug, AmB, was significantly enhanced in the presence of allicin, because of the similar mechanism of action of these two compounds. Therefore, the combination of allicin and an antifungal drug may be effective at treating fungal infections with minimal side-effects.

## Materials and Methods

### Culture and Preparation of *C. albicans*

*C. albicans* cells (ATCC, Rockville, MD, USA) were maintained in Sabouraud broth and incubated at 37°C for 24 hours in a shaking incubator at 180 rpm. The cells were centrifuged at 2500 rpm for 15 minutes and then washed in calcium-, magnesium-free phosphate-buffered saline (DPBS).

### Viability of *C. albicans*

Allicin was purchased from Allimax® (Allimax Nutraceuticals, Chicago, USA) at a guaranteed 100% yield of pure stabilized allicin extract. The minimum inhibitory concentrations (MIC_10_) of allicin were calculated by measuring cell viability at different concentrations of allicin ranging from 0 to 5 µg/mL. The MIC_10_ of the antifungal agents amphotericin B and flucytosine were determined according to the method of Brito et al. [19]. *C. albicans* were grouped by treatment conditions as follows: control, allicin, AmB, flucytosine, allicin + AmB, allicin + flucytosine, allicin + AmB + flucytosine. For all groups, cell viability was evaluated by Trypan blue staining after 0, 6, 12, 18, and 24 hours of treatment. Details of how the cell viability measurements were performed are described in a previous study [18].

### Clearing Assay

*Candida* cells (1×10^6^ cells/ml) were inoculated onto Sabouraud dextrose agar plates. Antifungal-containing discs (AmB, flucytosine, allicin, AmB + allicin, flucytosine + allicin, AmB + flucytosine + allicin) were introduced onto the plate at three different concentrations (MIC_10_×10^−1^, MIC_10_, and MIC_90_). The MIC_10_ and MIC_90_ of allicin were 1 µg/ml and 128 µg/ml, respectively [16]. The MIC_10_ and MIC_90_ of AmB and Flu were 0.1 µg/ml and 1 µg/ml, respectively [26]. Plates were incubated at 37°C for 24 hours, and then photographed.

### SEM and AFM Observations

*C. albicans* cells were fixed in 2.5% glutaraldehyde in 0.1 M PBS for 30 minutes and washed with 0.1 M PBS. To dehydrate the cells, collected cells were immersed in 100% ice-cold acetone for 10 minutes. For SEM measurements, *C. albicans* were smeared on a silver stub like a thin film, and the samples were gold-coated by cathodic spraying (Polaron gold). The SEM observations were made using a Cambridge Instruments S250 SEM. All observations were performed under the conditions of EHT = 20.00 kV, WD = 9.5 mm, Signal A = SE1. Nanoscale morphological changes and the biophysical properties of *C. albicans* were investigated by using an AFM system (Surface Imaging Systems, Herzogenrath, Germany). All images were measured in contact mode (Budget Sensor, Bulgaria) with a resolution of 256×256 pixels and a scan speed of 0.2 lines/sec. The probes used for imaging had a resonance frequency of 13 kHz (±4 kHz), a force constant of 0.2 N/m (±0.14 N/m), a cantilever length of 450 µm (±10 µm), a cantilever width of 38 µm (±5 µm), a cantilever thickness of 2 µm (±1 µm), a tip radius of 5 nm (±1 nm), and a tip height of 17 µm (±2 µm).

The stiffness and adhesive forces of the cells were determined by force-distance curve (FD) measurements. The FD curves were measured at the loading rate of 1 µm/s. The elasticity of *C. albicans* was calculated according to the equation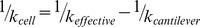 where *k*_effective_ and *k*_cantilever_ were determined from the slope of the linear region of approaching curve for *C. albicans* and a slide glass, respectively [27].
